# The impact of linguistic features on CTR in Instagram ads: A study of supplement and cosmetic products

**DOI:** 10.1371/journal.pone.0338313

**Published:** 2026-04-15

**Authors:** Kenjiro Inoue, Mitsuo Yoshida

**Affiliations:** 1 Degree Programs in Systems and Information Engineering, University of Tsukuba, Ibaraki, Japan; 2 Institute of Business Sciences, University of Tsukuba, Tokyo, Japan; Universiti Monash Malaysia: Monash University Malaysia, MALAYSIA

## Abstract

This study analyzes linguistic features impacting click-through rate (CTR) in Japanese Instagram ads (21,692 ads; July 2021-June 2023, Meta’s Marketing API). CTR was computed as link clicks/impressions from Meta’s Ads Manager. Using J-LIWC2015, we quantified psycholinguistic dimensions, predominantly in Japanese. Multivariate regression models, controlling for caption length, log-transformed impressions, and product-level fixed effects, identified distinct linguistic patterns predicting CTR by product category. For supplement ads, “risk” (β=0.108) and “discrepancy” (β=0.051) positively impacted CTR; “motion” (β=−0.090) and “negative emotion” (β=−0.076) decreased it. For cosmetic ads, “see” (β=0.134), “positive emotion” (β=0.088), and “motion” (β=0.046) were positive predictors, while “body” (β=−0.103) and “negative emotion” (β=−0.053) decreased it. These findings underscore the critical role of linguistic features in enhancing advertising impact when aligned with the psychological needs of target audiences. By leveraging these insights, marketers can develop data-driven communication strategies to optimize engagement on Instagram.

## Introduction

In the contemporary digital economy, online advertising has become a primary channel for reaching consumers, with social media platforms like Instagram commanding a significant share of the market [1]. The visual-centric nature of Instagram has led to a substantial body of research focused on the impact of image and video content on user engagement [2]. Similarly, the effectiveness of algorithmic targeting and personalization has been a major area of academic and industry focus [3–5]. However, a critical component of advertising effectiveness, the linguistic content of the ad copy itself, remains comparatively underexplored. This research gap is particularly pronounced for non-English languages and in non-Western cultural contexts, where linguistic norms and persuasive appeals may differ significantly. This study aims to bridge this gap by systematically investigating how specific linguistic features within Japanese-language Instagram ad copy impact a key performance metric: the click-through rate (CTR). We anchor our investigation in established theories of persuasion, primarily the Elaboration Likelihood Model (ELM) [6] and Framing Theory [7]. Recent work has explored ELM’s applicability in emerging contexts like virtual influencers and high/low-involvement products [8]. ELM posits that persuasion occurs via two distinct routes: a central route, characterized by careful and thoughtful consideration of the argument’s merits, and a peripheral route, which relies on heuristic cues such as source credibility or emotional appeals. Framing Theory suggests that the way information is presented—as either a potential gain or a potential loss—can significantly alter its persuasive impact. We hypothesize that the impact of these routes and frames is not universal but is contingent on the product category. Specifically, we examine two distinct but prominent product categories in online advertising: health supplements and cosmetics. We propose that “supplement” products, which relate to health and are often considered higher-involvement purchases, may be more susceptible to persuasion via the central route and loss-framing (e.g., avoiding a health risk). In contrast, “cosmetic” products, which are often tied to aspirational goals and aesthetics, may be more effectively promoted through the peripheral route and gain-framing (e.g., achieving a more beautiful appearance). To test these propositions, this study pursues three primary objectives:

1. To quantify the prevalence of various psycholinguistic features, as defined by the Japanese Linguistic Inquiry and Word Count (J-LIWC2015) dictionary [9], in a large corpus of Japanese Instagram ads for supplement and cosmetic products.
2. To determine which of these linguistic features significantly predict ad performance (CTR) after controlling for potential confounding factors like ad length and brand-level effects.
3. To formally test whether the impact of these linguistic features on CTR differs significantly between the two product categories.

**Fig 1:**
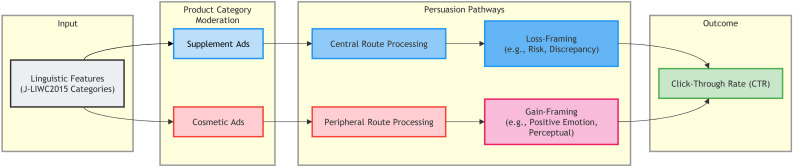
Conceptual model of linguistic feature impact on CTR. This diagram illustrates how specific LIWC categories are hypothesized to impact Click-Through Rate (CTR) via Elaboration Likelihood Model (ELM) routes and Framing Theory, contingent on product category.

By analyzing a large-scale, real-world dataset of 21,692 ads, this study makes a significant contribution to the literature on computational advertising and consumer psychology. It is one of the first studies to empirically link J-LIWC2015 categories to a behavioral outcome metric (CTR) in the Japanese digital advertising ecosystem. The findings provide actionable, data-driven insights for marketers seeking to optimize their ad copy and offer empirical evidence for the context-dependent nature of persuasion in online environments. The relationship between these theoretical frameworks and our linguistic variables is further illustrated in Figure 1. This conceptual model guides our investigation by positing that specific psycholinguistic features (LIWC categories) impact CTR through distinct ELM processing routes and framing orientations, depending on the product category.

## Background

### Linguistic features in advertising

The power of language in advertising is a foundational concept in marketing, dating back to early work on the psychology of selling [10]. While modern digital advertising research has often prioritized visual elements and targeting technology, the textual component remains a crucial interface for communicating value, evoking emotion, and prompting action [11]. Recent work highlights how influencer characteristics and live content impact impulsive buying in e-commerce [12]. Prior research has shown that specific word choices can significantly impact consumer attitudes and behaviors. For example, the use of emotional language can enhance ad effectiveness by creating a positive affective response, which then transfers to the brand itself [13]. Similarly, cognitive language that prompts analytical thought can be effective for products requiring more deliberate consideration [14]. However, much of this research has been conducted in controlled experimental settings or has focused on English-language advertising, leaving a gap in our understanding of these dynamics in large-scale, real-world, non-English environments. Table 1 summarizes seminal linguistic studies on online advertising.

**Table 1:** Prior linguistic studies on online advertising and their relevance. This table summarizes seminal studies investigating the impact of linguistic features on online ad performance. It outlines the authors, platforms, specific linguistic variables analyzed, key findings related to CTR or other performance metrics, and their direct relevance to the current study. The table highlights diverse approaches to text analysis in digital advertising, emphasizing how various linguistic elements contribute to consumer engagement across different contexts.

Author(s) and Year	Platform / Context	Linguistic Variables	Key CTR / Performance Findings	Relevance to Current Study
Richardson et al. (2007)	Search Ads (MSN)	Query–ad text match; URL	Direct text matches increase CTR	Early emphasis on textual relevance
Trofimov et al. (2012)	Online Ads	Emotions; readability metrics	Positive emotion and high clarity boost CTR	Shows affective language and processing‐fluency effects
Effendi (2017)	Contextual Ads	Keywords; sentiment score	Sentiment polarity and shorter words perform better	Highlights importance of word choice
Igarashi et al. (2021)	Japanese online communication	J-LIWC2015 categories	Empirically validates J-LIWC2015 lexical coverage	Core tool for Japanese language analysis
Pennebaker (2015)	General text	LIWC categories (social, cognitive, ...)	Language reflects psychological states	Foundational LIWC framework
Boyd (2022)	Social media	LIWC (authenticity, emotion, ...)	Language features linked to engagement	Demonstrates LIWC utility in social platforms

### Japanese cultural context and linguistic nuances in advertising

The effectiveness of linguistic strategies in advertising is deeply intertwined with cultural context. In Japan, communication often emphasizes subtlety, indirectness, and a high-context approach, contrasting with more direct communication styles prevalent in Western cultures [15]. This manifests in advertising through the nuanced use of language and non-verbal cues. For instance, while Western ads might employ direct emotional appeals, Japanese advertising frequently leverages rhetorical expressions like metaphor and metonymy to evoke feelings and convey product value indirectly through imagery and association, particularly in beauty and health sectors [15]. These approaches align with Japanese cultural values of harmony and implicit understanding.

Further, Japanese language boasts a rich vocabulary of onomatopoeic and mimetic expressions (known as EEEs - Embodied Emotional Expressions) which are intuitively used to convey nuanced emotions and sensations [16]. The pervasive “kawaii” (cute) culture also serves as a distinct persuasive strategy, where adorable characters and gentle language can bypass direct argumentation to create affective appeal and build trust [17]. Such culturally specific linguistic preferences underscore the importance of employing tools like J-LIWC2015, which is optimized for Japanese text, to accurately capture these psycholinguistic dimensions accurately. Moreover, in highly regulated sectors like health and cosmetics, Japanese advertising must navigate strict legal frameworks (e.g., Pharmaceutical and Medical Device Act, Act against Unjustifiable Premiums and Misleading Representations) that impact permissible linguistic claims, especially regarding health benefits and exaggerated expressions. Understanding these cultural and regulatory nuances is crucial for developing effective advertising strategies in the Japanese market.

### LIWC as a tool for psychological text analysis

To systematically analyze the psychological dimensions of language at scale, researchers have increasingly turned to computational text analysis tools. One of the most established and validated tools is the the Linguistic Inquiry and Word Count (LIWC) [18,19]. LIWC is a dictionary-based software that analyzes text by calculating the percentage of words that impact into psychologically meaningful categories. These categories include affective processes (e.g., positive and negative emotion), cognitive processes (e.g., insight, causation), social processes, and various content-related themes (e.g., health, money, risk). Unlike complex machine learning models that can be difficult to interpret, LIWC’s dictionary approach provides transparent and theoretically grounded metrics. It has been successfully applied across numerous domains to link language patterns to psychological states and real-world outcomes, such as predicting political elections from tweets [20] or assessing public opinion [21]. This study utilizes the officially validated Japanese version, J-LIWC2015 [9], to apply this robust methodology to Japanese advertising copy.

### Persuasion theories in advertising contexts

Our analysis is guided by two complementary theories of persuasion: the Elaboration Likelihood Model (ELM) [6] and Framing Theory [7]. ELM posits that persuasion occurs via two distinct routes: a central route, characterized by careful and thoughtful consideration of the argument’s merits, and a peripheral route, which relies on heuristic cues suchs as source credibility or emotional appeals. Framing Theory complements ELM by focusing on how information is presented [7]. A gain-frame impacts the positive outcomes of taking an action (e.g., “achieve radiant skin”), while a loss-frame impacts the negative consequences of not taking an action (e.g., “don’t let tired skin hold you back”). Research, including Protection Motivation Theory [22], suggests that for health-related behaviors, loss-framing can be particularly effective because it activates a desire to mitigate risk. Therefore, we expect that “supplement” ads may benefit from loss-framed language (e.g., LIWC categories like “risk”). Conversely, aspirational “cosmetic” products may benefit more from gain-framed language that highlights positive outcomes (e.g., LIWC categories like “posemo” and “achieve”). By integrating these theoretical frameworks, this study moves beyond simply identifying which words work, aiming to understand why they work in different product contexts.

## Methods

### Data collection

This study analyzes linguistic features impacting click-through rate (CTR) in Japanese Instagram ads (21,692 ads; July 2021-June 2023, Meta’s Marketing API). All data were collected from Meta’s Marketing API under standard developer agreements, ensuring compliance with their Terms of Service. Advertisements included were publicly accessible posts from Meta Business accounts. The anonymized dataset used in this study is available in the S1 File.

### Inclusion and exclusion criteria

To ensure the relevance and consistency of our dataset, we applied specific inclusion and exclusion criteria. Data were collected from Meta’s Marketing API, a repository of advertisements run on Meta’s platforms. The collection period spanned two years, from July 1, 2021, to June 30, 2023. Our initial query targeted ads delivered to users in Japan. From this initial collection, we implemented a multi-step filtering process to construct our final analytical dataset. We first identified and isolated ads belonging to two of the most frequently advertised product categories: “supplement” and “cosmetic”. The selection of “supplement” and “cosmetic” categories was based on their high frequency within our dataset and their theoretical relevance to the Elaboration Likelihood Model and Framing Theory. These two categories also represent psychologically and commercially distinct segments—health-related versus beauty-oriented products—making them a theoretically meaningful and practically relevant comparison for testing differential persuasion mechanisms.

As shown in Table 2, these two categories accounted for the largest number of total ads and products, making them ideal for a focused comparative analysis. We focused our analysis on ads predominantly written in Japanese, ensuring their relevance to J-LIWC2015. We defined ‘predominantly written in Japanese’ as ad captions where at least 30% of the tokens (after preprocessing) were matched to the J-LIWC2015 dictionary, which is optimized for Japanese text. The 30% threshold was chosen to balance linguistic coverage and sample retention, ensuring sufficient representation of Japanese text without excessively excluding ads containing mixed or code-switched language. We did not apply a fixed token-based language filtering threshold to retain the real-world linguistic diversity of Instagram ad copy. To ensure that the ad copy was substantial enough for meaningful linguistic analysis, we applied an inclusion criterion requiring a minimum caption length of 5 words. To ensure CTR reliability, ads with missing click data and those with fewer than 50 clicks were excluded from the analysis. While the exclusion of ads with fewer than 50 clicks improves the stability of CTR estimates, it may also introduce bias by disproportionately filtering out smaller advertisers, which is noted as a limitation of this dataset. We did not exclude duplicate ad creatives. This rigorous filtering process resulted in a final dataset of 21,692 unique advertisements. Each ad was assigned a unique product ID. The dataset was composed of 12,206 “supplement” ads and 9,486 “cosmetic” ads. For each ad, we retained the full text of the primary caption, along with the total number of impressions and clicks. The distribution of advertisements across all collected product categories is summarized in Table 2. This table clearly indicates that “supplement” and “cosmetic” categories represent the largest proportion of advertisements within our collected data, justifying their selection for focused analysis in this study. It should be noted that this study focuses solely on the linguistic content of advertisements and does not include audience-level or targeting-related variables such as demographic segmentation, device type, or delivery optimization, which may also influence CTR.

**Table 2:** Distribution of advertisements across product categories on Instagram. This table shows the total number of advertisements and the average, median, and maximum number of ads per product for each category. The data indicates that “supplement” and “cosmetic” categories account for the largest proportion of advertisements.

Category	Products	Total Ads	Ads per Product		
			Mean	Median	Max
Supplement	61	12,206	200	58	2,483
Cosmetic	51	9,486	185	86	1,898
Food	5	1,421	284	278	535
Online Education	3	421	149	149	222
Clinic	2	3,414	1,707	1,707	2,658
Oral Care	2	13	7	7	9
Dating	1	793	793	793	793
Clothing	1	625	625	625	625
Genetic Testing	1	546	546	546	546
Event Promotion	1	421	421	421	421
Pets	1	2	2	2	2

### Ethical considerations

This study analyzed secondary, non-identifiable advertising data obtained under a standard developer agreement with Meta. Under the authors’ institutional guidelines, research that involves no interaction with human subjects and uses only de-identified data is exempt from Institutional Review Board oversight; therefore, no ethical approval was required. All data were collected from Meta Marketing API. The dataset used in this study is anonymized and contains no personally identifiable information. A critical requirement for this study is compliance with data source policies. All data collection and analysis strictly adhered to Meta’s Marketing API Terms of Service and Platform Policies, ensuring the proper use and handling of publicly accessible advertising data.

### Linguistic feature extraction

**Fig 2:**
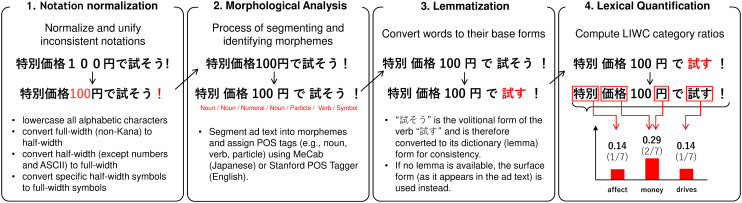
Overview of the J-LIWC2015 preprocessing pipeline for ad texts. This flowchart illustrates the four key steps involved in preparing Instagram ad texts for J-LIWC2015 analysis: 1) Notation normalization to unify linguistic variations, 2) Morphological analysis to segment Japanese sentences into morphemes, 3) Lemmatization to convert words to their base forms for accurate dictionary matching, and 4) Lexical quantification to compute the proportion of words belonging to each LIWC category.

We conducted linguistic preprocessing and feature extraction using the J-LIWC2015 dictionary. All ad texts were tokenized using MeCab with the IPA dictionary, and the frequency of each LIWC category was computed. Stopwords and platform-specific tokens such as hashtags and URLs were excluded prior to analysis. An overview of the preprocessing and feature extraction workflow is shown in Figure 2. We used word count as a control variable to account for message length. Full preprocessing details, including normalization schemes and illustrative tokenization examples, are provided in S1 Text (see S2 File).

### Measures

The primary outcome variable was the click-through rate (CTR), a standard indicator of user engagement in digital advertising. CTR was calculated for each advertisement as (Total Clicks / Total Impressions) × 100. CTR was treated as a continuous variable because it represents proportional engagement rather than a binary outcome, and modeling it continuously preserves more information about the variance in advertising performance. Given the typically skewed distribution of CTR, a logarithmic transformation $\log_{10}(\text{CTR}+0.0001)$ was applied to approximate normality for linear regression. The small constant ensured that ads with zero CTR were retained in the analysis. The main predictor variables were the J-LIWC2015 category scores, representing the percentage of caption words matching each psycholinguistic category. All LIWC scores were standardized as z-scores (mean = 0, standard deviation = 1) to enable comparability of effect sizes across categories. Two covariates were included to isolate the linguistic effects. First, caption word count was controlled for to account for variation in message length. Second, product-level fixed effects were implemented using dummy variables for each product ID, controlling for unobserved, time-invariant characteristics such as brand equity, pricing, visual design, and targeting configuration.

### Research design and statistical analysis

This study employs a quantitative, cross-sectional design to examine the relationship between linguistic features and advertising performance. Specifically, we address two primary research questions for Instagram advertisements within the “supplement” and “cosmetic” product categories:

- **RQ1:** Do psycholinguistic features in Japanese Instagram ad copy predict click-through rates (CTR)?
- **RQ2:** Does the predictive power of these psycholinguistic features differ between “supplement” and “cosmetic” product categories?

To answer these questions, we constructed a series of multivariate Ordinary Least Squares (OLS) regression models. Model 1 assessed the continuous effect of linguistic intensity by regressing log-transformed CTR on the standardized proportion of words in each J-LIWC2015 category. Separate models were estimated for each product type. The specification was:


$$\log({\text{CTR}}_{i})=\beta_{0}+\sum_{k=1}^{K}\beta_{k}({\text{LIWC Proportion}}_{ki})+\gamma ({\text{WordCount}}_{i})+\alpha_{p}+\epsilon_{i},$$

where *i* indexes the advertisement, *k* indexes LIWC categories, $\alpha_{p}$ denotes product-level fixed effects, and $\epsilon_{i}$ is the error term. Model 2 focused on threshold effects, using binary indicators of LIWC category presence as predictors, while retaining the same structure as Model 1. To investigate RQ2 directly, we also estimated models using the full dataset (N = 21,692) that included interaction terms between each LIWC feature and a product category dummy (1 = “cosmetic”, 0 = “supplement”):


$$\log({\text{CTR}}_{i})=\beta_{0}+\beta_{1}({\text{LIWC}}_{i})+\beta_{2}({\text{Category}}_{i})$$

$$+\beta_{3}({\text{LIWC}}_{i}\times {\text{Category}}_{i})+\text{Controls}+\alpha_{p}+\epsilon_{i}.$$

A significant interaction coefficient ($\beta_{3}$) indicates differential effects of linguistic features by category.

To assess multicollinearity, we calculated Variance Inflation Factors (VIFs) for all predictors. To avoid redundancy from hierarchical LIWC categories, we excluded parent-level constructs and retained only subcategories. Maximum VIFs were 2.78 for Models 1 and 2 and 4.12 for interaction models, all below the threshold of 5. Robustness was verified through additional logistic regressions using a dichotomized CTR: ads in the top 25th percentile (CTR > 0.985%) were coded as 1 (“high CTR”), others as 0 (“low CTR”). This analysis serves as a robustness check, confirming that the key findings are not dependent on the choice of the regression model. Results largely confirmed the direction and statistical significance of the coefficients for the primary linguistic predictors observed in the OLS models. For supplements, “risk” and “discrepancy” retained positive significance; for cosmetics, “see”, “positive emotion”, and “motion” were positive predictors, while “body” and “negative emotion” had negative effects. These results, including coefficients and p-values, are detailed in S1 Appendix (Tables 5 and 6). Effects remained stable after adjusting for word count and log-impressions. Finally, to correct for multiple testing across LIWC categories, we applied the Benjamini-Hochberg procedure with a 5% false discovery rate. This adjustment mitigates the risk of Type I errors and improves the reliability of the reported findings.

## Results

### Descriptive statistics

**Fig 3:**
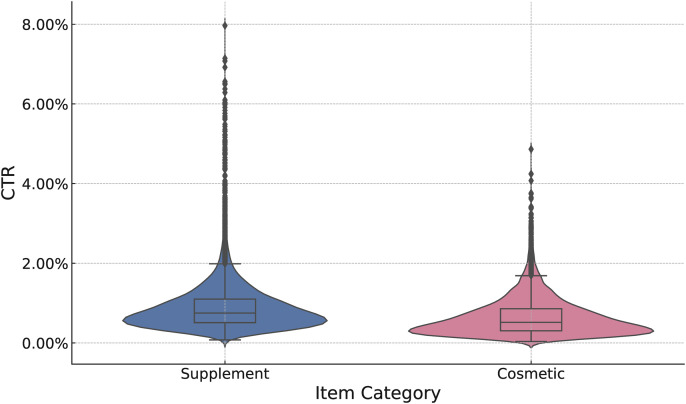
Violin plots showing the distribution of CTR for “supplement” and “cosmetic” categories. The figure demonstrates that “supplement” advertisements impact a broader variability in CTR compared to “cosmetic” advertisements.

A descriptive analysis of the J-LIWC2015 categories showed notable differences in the language typically used for each product type (Table 3; see also Figure 3). Supplement advertisements, on average, contained a higher proportion of words related to “Drives” (e.g., achievement, reward), “Relativity” (e.g., time, space), and, most notably, “Risk”. In contrast, cosmetic advertisements featured a significantly higher prevalence of words related to “Affective Processes” (especially “Positive Emotion”) and “Perceptual Processes” (especially “See”), indicating a greater focus on emotional appeal and visual outcomes.

**Table 3:** Descriptive statistics of key LIWC categories. This table presents the mean and standard deviation of the percentage of words per ad for key J-LIWC2015 categories, separated by product category (Supplement and Cosmetic).

LIWC Category	Supplement (N = 12,206)		Cosmetic (N = 9,486)	
	Mean	SD	Mean	SD
Affective Processes	3.15	2.50	4.21	3.10
Positive Emotion	2.55	2.10	3.65	2.80
Negative Emotion	0.60	1.25	0.56	1.15
Cognitive Processes	5.50	3.40	5.10	3.20
Discrepancy	0.45	0.90	0.35	0.80
Perceptual Processes	2.10	2.00	3.50	2.60
See	1.20	1.50	2.40	2.10
Drives	6.80	3.80	6.50	3.90
Risk	0.95	1.40	0.25	0.60
Motion	0.80	1.10	0.90	1.20

### RQ1: Linguistic predictors of CTR by category

**Fig 4:**
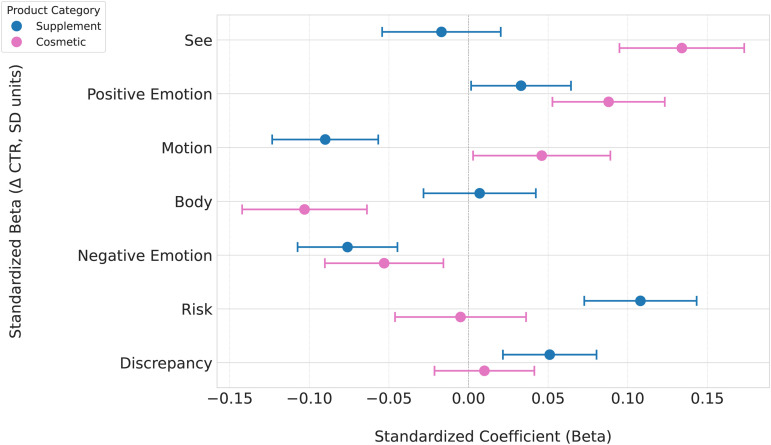
Standardized coefficients (Beta) of key linguistic features on CTR. This forest plot displays the standardized regression coefficients (*β*) and 95% confidence intervals for the effect of each J-LIWC2015 category on log-transformed CTR, separated by product category (Supplement vs. Cosmetic).

The regression analyses identified several significant linguistic predictors of CTR for each product category, after controlling for word count and product-level fixed effects (Table 4 and Figure 4). For supplement advertisements (Model 1), the strongest positive predictor of CTR was the “Risk” category (β=0.108), indicating that ad copy impacting health risks or concerns was highly effective at driving engagement. The “Discrepancy” category, which includes words that highlight a gap between a current and desired state (e.g., “lack”, “should”), was also a significant positive predictor (β=0.051). Conversely, words related to “Motion” (β=−0.090) and “Negative Emotion” (β=−0.076) were negatively associated with CTR.

**Table 4:** OLS regression results: Linguistic features impacting log-transformed CTR. This table presents standardized coefficients (*β*) from three OLS regression models. Model 1 (Supplement) and Model 2 (Cosmetic) show category-specific effects. The pooled interaction model identifies significant differences in linguistic effects by product category. All models control for word count and product-level fixed effects. Significance (Benjamini-Hochberg FDR corrected q-values) is indicated. Standard errors are in parentheses for the pooled interaction model. Full model fit statistics (R^2^, Adj R^2^, AIC, BIC) are provided, including null model benchmarks for comparison.

	Model 1	Model 2	Pooled (Interaction)
Variable	Supplement	Cosmetic	Model (Interaction)
*(Main Effects)*			
Risk	0.108***	−0.005	0.107***
	—	—	(0.018)
Discrepancy	0.051***	0.010	0.050***
	—	—	(0.015)
See	−0.017	0.134***	−0.014
	—	—	(0.019)
Positive Emotion	0.033*	0.088***	0.033*
	—	—	(0.016)
Motion	−0.090***	0.046*	−0.086***
	—	—	(0.017)
Body	0.007	−0.103***	0.004
	—	—	(0.018)
Negative Emotion	−0.076***	−0.053**	−0.076***
	—	—	(0.016)
*(Interaction Effects)*			
Cosmetic (Category Dummy)			−1.309***
			(0.035)
Risk × Cosmetic			−0.113***
			(0.028)
See × Cosmetic			0.149***
			(0.027)
Motion × Cosmetic			0.136***
			(0.028)
Body × Cosmetic			−0.118***
			(0.027)
Word Count	Yes	Yes	Yes
Product Fixed Effects	Yes	Yes	Yes
Observations	12,206	9,486	21,692
R^2^	0.231	0.217	0.245
Adjusted R^2^	0.231	0.217	0.245
AIC	−2944.40	−2120.57	−5540.67
BIC	−2012.33	−1407.41	−4021.46

*Standard errors in parentheses are for the pooled interaction model only.*

*q < 0.05,  **q < 0.01,  ***q < 0.001 (Benjamini-Hochberg correction).

*Observations: Model 1 = 12,206;  Model 2 = 9,486;  Pooled (Interaction) Model = 21,692.*

*Adjusted R* ^*2*^ *: Model 1 = 0.231;  Model 2 = 0.217;  Pooled (Interaction) Model = 0.245.*

*R*^*2*^ *(Null): Model 1 = 0.000;  Model 2 = 0.000;  Pooled (Interaction) Model = 0.000.*

*AIC (Null): Model 1 = -2908.57;  Model 2 = -2077.51;  Pooled (Interaction) Model = -5432.86.*

*BIC (Null): Model 1 = -2822.42;  Model 2 = -2004.99;  Pooled (Interaction) Model = -5364.50.*

For cosmetic advertisements (Model 2), a different set of features emerged as dominant. The strongest positive predictor was the “See” category (β=0.134), which includes words related to visual appearance (e.g., “shine”, “bright”, “flawless”), strongly supporting the idea that visual and aesthetic benefits are key drivers of engagement. The “Positive Emotion” category (β=0.088) also showed a statistically significant positive association. Interestingly, and in direct contrast to supplements, “Motion”-related words were positively associated with CTR in this context (β=0.046). Language that directly referenced the “Body” (β=−0.103) was negatively associated with CTR. The “Negative Emotion” category (β=−0.053) was also negatively associated with CTR. These results provide a direct answer to RQ1, demonstrating that specific psycholinguistic features in Japanese Instagram ad copy significantly predict CTR, and these effects vary distinctly across product categories. The results highlight a distinct contrast in persuasive strategies: while supplements benefit from language addressing risks and discrepancies, cosmetics thrive on visual and emotional appeals. This stark difference underscores the category-specific nature of effective ad copy.

### RQ2: Interaction between language and product category

The pooled regression model (referred to as the interaction model in Table 4) confirmed that the impacts of several key linguistic features were statistically different across the two product categories, as supported by a joint Wald test ($\chi^{2}=111.46$, df = 7, p < 0.001). The interaction term “Risk x Cosmetic” was significantly negative (β=−0.113,q<0.001), indicating that the positive impact of risk-related language on CTR was significantly weaker for cosmetics compared to supplements. Conversely, the interaction term “See x Cosmetic” was significantly positive (β=0.149,q<0.001), confirming that the positive impact of visual, perceptual language was significantly stronger for cosmetics. Most strikingly, the “Motion x Cosmetic” interaction was large and highly significant (β=0.136,q<0.001), formally demonstrating that the impact of motion-related words reverses direction between the two categories: it is detrimental for supplement ads but beneficial for cosmetic ads. The interaction term “Body x Cosmetic” was also significant and negative (β=−0.118,q<0.01), indicating that the impact of “Body” language observed in cosmetic ads was significantly weaker (or negative) when compared to supplements (where “Body” was not a strong predictor). These significant interaction effects provide strong evidence that the optimal linguistic strategy for driving user engagement is highly dependent on the product being advertised. The significant interaction effects observed in this model directly address RQ2, providing empirical evidence that the impact of linguistic features on CTR is contingent on the product category. This finding reveals a key reversal in linguistic effectiveness: language that is negatively associated with CTR for supplement ads can be highly beneficial for cosmetic ads, and vice versa. This provides strong empirical support for the theoretical proposition that optimal linguistic strategies are context-dependent.

## Discussion

This study’s findings suggest that the linguistic content of Instagram advertisements is a significant predictor of user engagement, with its impact notably moderated by product category. The results indicate two potentially distinct persuasive pathways for “supplement” and “cosmetic” products, which can be interpreted through the theoretical lenses of the Elaboration Likelihood Model (ELM) and Framing Theory. Our work extends these established frameworks by providing empirical evidence from a large-scale, non-Western context, demonstrating how specific psycholinguistic features activate distinct central/peripheral processing routes and loss/gain framing orientations depending on the product category.

For supplement products, the data appear to be consistent with a persuasion model based on central-route processing and loss-framing. The prominence of “Risk” as a positive predictor of CTR aligns with Protection Motivation Theory. The “Discrepancy” category also showed a statistically significant positive association, suggesting a persuasive strategy that highlights gaps between current and ideal states. This type of messaging encourages a deliberate, cognitive evaluation of the product as a solution, characteristic of the central route to persuasion. In contrast, for cosmetic products, results suggest a more reward-seeking and aspirational persuasion model. The strong positive impact of “See” (perceptual) is consistent with an emphasis on aesthetic appeal, while “Positive Emotion” also showed a significant positive association. These findings highlight a clear divergence in persuasive appeals, with supplements leveraging cognitive, problem-solving language and cosmetics relying on visual and emotional cues to drive engagement. Language directly referencing the “Body” was negatively associated with CTR, possibly indicating that explicit mentions of physical flaws or discomfort may deter engagement. Interestingly, and reversing its effect in supplements, “Motion” words were positively associated with CTR for cosmetics; this could support an interpretation where action-oriented language frames the product as part of a dynamic, aspirational lifestyle, serving as an attractive peripheral cue. This reversal may reflect a difference in the underlying motivational orientation of consumers. For cosmetics, motion-related language may resonate with approach-oriented goals such as self-enhancement and transformation, whereas for supplements, similar expressions might imply physical strain or risk, leading to avoidance-oriented interpretations. The “Negative Emotion” category was also negatively associated with CTR, implying it can deter clicks in this context as well.

The significant interaction effects provide empirical support for the core theoretical argument of this paper: that a one-size-fits-all approach to crafting effective ad copy may be suboptimal. The findings suggest that the optimal linguistic strategy is likely contingent on the consumer’s psychological mindset, which seems to be intrinsically linked to the product category. Supplements appear to appeal to a problem-solving, risk-averse mindset, whereas cosmetics may resonate with an aspirational, reward-seeking one.

From a managerial perspective, these findings offer actionable, data-driven insights that can inform ad-copy optimization for digital marketers and copywriters. To improve effectiveness, advertising language could be tailored to the psychological motivations associated with each product category. For supplement products, ad copy could be framed to present the product as a concrete solution to health-related concerns or as a tool to bridge a wellness gap. Language that evokes a sense of urgency or protection appears to be effective. For example, rather than using generic phrases such as “Feel great,” more impactful language might be “Don’t let fatigue compromise your health—reduce your risk with our daily supplement.” The observed positive effect of discrepancy-related language further supports this potential strategy. For cosmetic products, marketers could benefit from prioritizing sensory and visually descriptive language, as words associated with perceptual processes (e.g., “See”) were among the strongest predictors of CTR in this category. While positive emotional language also demonstrated a significant effect, its impact was smaller in comparison, suggesting it may serve a complementary role. Additionally, dynamic and action-oriented language (e.g., “Motion”) can help frame the product as part of an aspirational lifestyle. In contrast, language that directly references the physical body was negatively associated with CTR, indicating that overt references to physical flaws may deter engagement. Instead, messages should focus on positive and aspirational outcomes. Finally, the use of negatively valenced language might be approached with caution. For supplements, narrowly framed risk language can be effective, but our findings suggest that broader expressions of negative emotion may reduce CTR. Similarly, for cosmetics, negative emotional cues also appear to decrease engagement. Together, these principles suggest the value of A/B testing different linguistic frames and highlight the potential need for context-specific experimentation to optimize ad performance.

The potential power of persuasive language, particularly fear- and risk-based appeals, warrants a discussion of ethical responsibilities. While our findings indicate that risk-framing can increase engagement for supplement ads, marketers have a responsibility to avoid overstating risks or making unsubstantiated health claims, which can mislead consumers and generate undue anxiety. Regulatory bodies and platform policies already place restrictions on health-related advertising, and our findings reinforce the importance of these guidelines. We believe effective marketing should be built on transparency and accuracy, using persuasive frames to highlight genuine benefits rather than to exploit consumer vulnerabilities. In this regard, ethical advertising practice requires aligning persuasive strategies with public health interests, ensuring that data-driven insights are used to inform responsible communication rather than to manipulate consumer behavior.

Unlike prior LIWC-based advertising studies conducted primarily in English and on Western platforms, this research extends psycholinguistic text analysis to a large-scale, non-English corpus within the Japanese advertising ecosystem. By doing so, it demonstrates the cross-cultural generalizability of LIWC constructs while also revealing culturally contingent linguistic effects that have not been documented in Western contexts. Furthermore, as digital platforms increasingly rely on algorithmic targeting, our findings underscore the importance of consumer protection and ethical data use in optimizing persuasive communication. Methodologically, this study demonstrates the feasibility of scalable psycholinguistic analysis in a non-English context, offering a framework that can be extended to other languages and markets.

In sum, this study provides large-scale empirical evidence that bridges computational psycholinguistics with digital marketing strategy, demonstrating that effective ad language depends on both psychological appeal and product category. By applying J-LIWC2015 to a large corpus of over 21,000 Japanese ads, our work offers a scalable and theoretically-grounded methodology for optimizing ad copy in diverse cultural contexts.

### Limitations and future research

This study is subject to several limitations that also present opportunities for future research. The most significant limitation is that our analysis relied solely on J-LIWC2015, which quantifies psycholinguistic dimensions based on tokenized words. As a dictionary-based approach, it can miss contextual nuance (e.g., irony, sarcasm), which may attenuate or obscure certain effects. This lexical scope means our models inherently exclude persuasive cues conveyed through multimodal signals, such as emojis and images, as well as instances of English code-switching within Japanese captions. Future studies should integrate computer-vision features or cross-lingual embeddings to enrich the understanding of multimodal persuasion. Another key limitation is that our current analysis does not account for specific audience characteristics, such as age, gender, or interests. Future studies would benefit from incorporating audience-level data to test for interaction effects between linguistic features and user profiles. Lastly, the cross-sectional nature of our data limits our ability to infer causality. Longitudinal studies or controlled experiments would be valuable for establishing causal relationships between linguistic features and advertising effectiveness over time.

## Conclusion

This study provides large-scale, empirical evidence that the linguistic features of ad copy are a critical driver of user engagement on Instagram, demonstrating that the path to persuasion is not universal but is contingent on the product category. It addresses both our research questions: for health supplements, language that addresses risk or discrepancy is most effective, while for cosmetics, language that evokes positive emotions and appeals to visual outcomes drives the highest engagement. These findings not only contribute to our theoretical understanding of persuasion in digital environments but also offer actionable guidance for marketers to create more resonant and effective advertising in a crowded digital marketplace.

## Supporting information

S1 Appendix Appendix A: Robustness analyses. (DOCX)

S1 File Instagram advertising dataset. This dataset contains anonymized caption texts, product category labels (supplement or cosmetic), impressions, clicks, and computed CTR values used in the analyses. Brand identifiers and sensitive information have been masked to comply with privacy requirements.
(CSV)

S2 File Text preprocessing details. This document provides full details of the preprocessing pipeline applied to Japanese Instagram ad captions, including normalization, morphological analysis, lemmatization, removal of non-linguistic elements, and lexical quantification using J-LIWC2015. The procedures correspond to the linguistic feature extraction methods described in the main text.
(DOCX)
