# Supplementary material for: The impact of linguistic features on CTR in Instagram ads: A study of supplement and cosmetic products
Source: PLoS One. 2026 Apr 15;21(4):e0338313. doi: 10.1371/journal.pone.0338313 (PMC13082717; doi:10.1371/journal.pone.0338313)
Supplement: S1 Appendix — (DOCX) [file pone.0338313.s001.docx]

**Appendix**

Appendix A: Robustness Analyses

This appendix provides detailed results of the robustness checks conducted to ensure the stability of our main findings across different model specifications.

Table 5. Logistic regression results: High CTR prediction for supplement advertisements. this table presents the logistic regression coefficients, standard errors, and Benjamini-Hochberg (BH) false discovery rate (FDR) corrected p-values for key linguistic features predicting high click-through rate (CTR) in supplement ads. High CTR is defined as the top 25th percentile of observed CTR values. The model controls for advertisement word count and product-level fixed effects. This analysis serves as a robustness check, confirming the consistent direction and significance of primary linguistic impacts observed in the main OLS models.

| LIWC Category | Coefficient (logit) | Std. Error | p-value |
| --- | --- | --- | --- |
| *Positive Predictors* |  |  |  |
| Risk | 0.251 | 0.035 | <0.001*** |
| Discrepancy | 0.112 | 0.029 | <0.001*** |
| Space | 0.089 | 0.027 | 0.001** |
| *Negative Predictors* |  |  |  |
| Motion | -−0.180 | 0.033 | <0.001*** |
| Negative Emotion | -−0.165 | 0.031 | <0.001*** |
| Causation | -−0.098 | 0.030 | 0.001** |

*Dependent Variable: High CTR (1 = Top 25th Percentile, 0 = Otherwise).*

*Standard errors in parentheses. All LIWC variables are standardized.*

**** p<0.001, ** p<0.01, * p<0.05 after Benjamini-Hochberg correction.*

Table 6. Logistic regression results: High CTR prediction for cosmetic advertisements. This table presents the logistic regression coefficients, standard errors, and Benjamini-Hochberg (BH) false discovery rate (FDR) corrected p-values for key linguistic features predicting high click-through rate (CTR) in cosmetic ads. High CTR is defined as the top 25th percentile of observed CTR values. The model controls for advertisement word count and product-level fixed effects. This analysis serves as a robustness check, confirming the consistent direction and significance of primary linguistic impacts observed in the main OLS models.

| LIWC Category | Coefficient (logit) | Std. Error | p-value |
| --- | --- | --- | --- |
| *Positive Predictors* |  |  |  |
| See (Perception) | 0.285 | 0.041 | <0.001*** |
| Positive Emotion | 0.190 | 0.037 | <0.001*** |
| Leisure | 0.125 | 0.035 | <0.001*** |
| midrule *Negative Predictors* |  |  |  |
| Body | -−0.210 | 0.039 | <0.001*** |
| Anxiety | -−0.115 | 0.033 | 0.001** |
| Negative Emotion | -−0.053 | 0.019 | <0.01** |
| Money | -−0.080 | 0.031 | 0.010* |

*Dependent Variable: High CTR (1 = Top 25th Percentile, 0 = Otherwise).*

*Standard errors in parentheses. All LIWC variables are standardized.*

**** p<0.001, ** p<0.01, * p<0.05 after Benjamini-Hochberg correction.*
