# Supplementary material for: The impact of linguistic features on CTR in Instagram ads: A study of supplement and cosmetic products
Source: PLoS One. 2026 Apr 15;21(4):e0338313. doi: 10.1371/journal.pone.0338313 (PMC13082717; doi:10.1371/journal.pone.0338313)
Supplement: S2 File — This document provides full details of the preprocessing pipeline applied to Japanese Instagram ad captions, including normalization, morphological analysis, lemmatization, removal of non-linguistic elements, and lexical quantification using J-LIWC2015. The procedures correspond to the linguistic feature extraction methods described in the main text. (DOCX) [file pone.0338313.s003.docx]

**S1 Text**

This document provides a detailed description of the text preprocessing pipeline applied to Japanese Instagram ad captions before linguistic feature extraction using the Japanese Linguistic Inquiry and Word Count 2015 (J-LIWC2015) dictionary.

**Step 1: Notation Normalization**

All ad captions were first normalized to unify orthographic conventions across Japanese and alphanumeric expressions. The following conversions were applied systematically:

- Full-width alphanumeric characters (Ａ～Ｚ, ａ～ｚ, ０～９) were converted to half-width.
- Punctuation marks and spacing were standardized.
- Roman letters were converted to lowercase.
- Variant forms of long vowels (ー／～) were retained in their original usage to preserve expressive intent.

This step ensured that equivalent expressions written in different character forms were treated consistently during tokenization.

**Step 2: Morphological Analysis**

Tokenization was performed using MeCab v0.996 with the IPA dictionary, which provides high-accuracy segmentation of Japanese text that lacks explicit word delimiters.

This process separated sentences into morphemes (the smallest grammatical units).

For example:

- 特別価格１００円で試そう！ → 特別 / 価格 / １００円 / で / 試そう / ！

This allowed for the accurate identification of individual lexical units, which were then passed to subsequent steps for lemmatization and dictionary matching.

**Step 3: Lemmatization**

Each token was converted to its dictionary base form (lemma) to ensure consistent matching with J-LIWC2015 categories.

For instance, inflected verbs such as “食べた” (ate) were converted to “食べる” (eat).

This process reduced redundancy arising from conjugational variants and enhanced the accuracy of lexical categorization.

**Step 4: Removal of Non-Linguistic Elements**

Non-verbal or platform-specific tokens—including URLs, hashtags, emojis, and user mentions—were removed prior to LIWC analysis.

Punctuation marks and numerals were retained for tokenization integrity but excluded from J-LIWC2015 scoring.

This ensured that only linguistically meaningful text contributed to the psycholinguistic metrics.

**Step 5: Lexical Quantification**

Following lemmatization, each token was matched to the corresponding categories in J-LIWC2015.

The frequency of words belonging to each category was divided by the total word count of the caption to compute the proportion (percentage) per LIWC category.

This yielded standardized linguistic feature scores for statistical analysis.

Handling of Word Count and Character Count

Two different length metrics were computed:

- **Character count:** total number of Japanese and alphanumeric characters in the caption.
- **Word count:** total number of tokens after morphological analysis.

Only word count was used as a control variable in the regression models to account for message length variability.

**Summary**

This preprocessing pipeline ensured that all Japanese ad captions were analyzed in a linguistically consistent and reproducible manner.

The process standardized orthography, isolated morphemes, and aligned lexical items with the J-LIWC2015 categories.

By following this approach, the linguistic variables used in our regression analyses accurately captured the psycholinguistic characteristics of Japanese advertising language.

**Correspondence to Main Text**

A concise summary of this pipeline is presented in the *Methods* section (Linguistic Feature Extraction subsection) of the main article.

Readers are referred to this Supporting Information (S1 Text) for full procedural details, including normalization schemes and tokenization examples.
